# Elucidating the role of *SHROOM4* in non-small cell lung cancer: expression patterns, clinical correlations, and potential functions

**DOI:** 10.3389/fimmu.2025.1723844

**Published:** 2026-01-07

**Authors:** Yuqi Zhang, Rong Qiang, Mengyang Ding, Lin Wang

**Affiliations:** 1 Center of Medical Genetics, Northwest Women’s and Children’s Hospital, Xi’an, Shaanxi, China; 2 Institute of Molecular Enzymology, School of Life Sciences, Suzhou Medical College, Soochow University, Suzhou, China

**Keywords:** angiogenesis, non-small cell lung cancer (NSCLC), *SHROOM4*, tumor microenvironment (TME), Wnt signaling

## Abstract

**Background:**

Lung cancer remains the leading cause of cancer-related mortality worldwide, with non-small cell lung cancer (NSCLC) being the most common type. *SHROOM4*, a protein integral to cytoskeletal organization and cellular signaling, has not been extensively studied in NSCLC.

**Methods:**

Through bioinformatics analysis of public databases, we investigated the expression of *SHROOM4* and its relationship with clinical outcomes and potential mechanism. And we validated the mRNA and protein expression of *SHROOM4* in lung squamous cell carcinoma (LUSC) tissues and corresponding normal tissues.

**Results:**

Our analysis demonstrated a notable downregulation of *SHROOM4* mRNA and protein expression, along with its high diagnosis capability in lung cancer, especially pronounced in LUSC, Additionally, higher levels of *SHROOM4* were linked to worse clinical outcomes in lung cancer, characterized by reduced survival and more advanced disease stages. Single-cell RNA-seq data and differential analysis show *SHROOM4’s* high expression in stromal cells and its association with angiogenesis and Wnt/Beta-Catenin pathways possibly through *ANGPTL7/SFTPC*. Meanwhile, *SHROOM4* was found to co-express with *PTPN13/CACNA1C* impacting the tumor microenvironment (TME) and to participate in critical signaling pathways like cell circle and WNT. Moreover, positive correlations were discovered between *SHROOM4* expression and immune infiltration scores in NSCLC.

**Conclusion:**

These results underscore the potential of *SHROOM4*, an anticancer role, as both a diagnostic and therapeutic target, particularly in LUSC. And *SHROOM4* may modulate NSCLC progression by affecting the TME in many ways. Further studies are essential to elucidate SHROOM4’s role in lung cancer progression and to validate its clinical utility.

## 1 Introduction

Lung cancer is the leading cause of cancer-related mortality globally, with non-small cell lung cancer (NSCLC) accounting for approximately 80-85% of all cases (1, 2). Among NSCLC subtypes, lung adenocarcinomas (LUAD) represent 50-60% of NSCLC, while lung squamous cell carcinomas (LUSC) account for 20-30% (3). Currently, despite significant advancements in diagnostic and therapeutic approaches, NSCLC is frequently diagnosed at an advanced stage, contributing to a poor prognosis for most patients (4). The 5-year survival rate was 56.3% for patients with stage I lung cancer, whereas it drops drastically to 4.7% for those diagnosed with stage IV disease (5). Approximately 60% of patients with advanced NSCLC subtypes have molecular changes that may be suitable for targeted therapy (6). Whereas, the changes and roles of Shroom family molecular in NSCLC patients is unclear nowadays.

The Shroom family members, containing SHROOM1, SHROOM2, SHROOM3 and SHROOM4, have recently garnered attention due to their critical roles in cell morphology, cytoskeletal organization, and cellular signaling (7). *SHROOM4* (gene ID # 57477), located on the X chromosome at Xp11.22, encodes a protein that is part of the APX/SHROOM family, known to regulate cytoskeletal rearrangements and cellular motility through a N-terminal PDZ domain, a coiled-coil structure, and a C-terminal ASD2 motif (8, 9). In normal human tissues, *SHROOM4* is most highly expressed in lung tissue and least expressed in pancreatic tissue (10). While SHROOM4 has been primarily studied in the context of Stocco dos Santos syndrome (OMIM #30034) (11, 12), its role and mechanisms in cancer biology remain underexplored. Abnormal expression of *SHROOM4* was reported in some cancers (13, 14). However, its specific expression patterns in lung cancer tissues and its functional contributions to LUSC progression have not been systematically investigated.

In this study, a subsequently analysis was applied to clarified the role of *SHROOM4* in NSCLC leveraging multiple online publicly available datasets. We analyzed in detail the gene and phosphorylation expression patterns of *SHROOM4* in NSCLC, particularly its potential functions, mutation status, immunity and distinct prognostic values of *SHROOM4* in lung cancer. Our results were anticipated to provide a potential candidate method in NSCLC for future diagnosis and targeted treatment.

## 2 Methods

### 2.1 Public database analysis of *SHROOM4* family genes expression differences in lung cancer

Raw data of pan-cancer, lung cancer (LUAD & LUSC), and adjacent cancer samples were downloaded from the TCGA database (https://portal.gdc.cancer.gov/ (accessed on 1 August 2023)) and UCSC Xena (https://xenabrowser.net/datapages/ (accessed on 1 August 2023)). Among them, there are 598 cases of adenocarcinoma samples, 50 adjacent cancer samples; 551 cases of squamous cell carcinoma samples, and 49 adjacent cancer samples. The RNAseq results were converted into log2 [TPM + 1] data format, and R language (v.4.2.1) was used for further analysis of *SHROOM 1–4* expression differences in lung cancer. The Proteomics module of the UCLCAN database (https://ualcan.path.uab.edu/) was used to analyze the differential expression of SHROOM4 protein in 10 common tumors and the expression differences of *SHROOM4* phosphorylation modification sites in LUSC and LUAD (15). The Kaplan-Meier Plotter database (https://kmplot.com/analysis/) were used to analyze the expression of *SHROOM4* in lung cancer pathological tissue samples and the difference in patient survival periods (16). The single-cell RNA sequencing (scRNA-seq) dataset was obtained from the NSCLC_EMTAB6149 database, containing immune, malignant, stromal, and other cell types from non-small cell lung cancer (NSCLC) samples. Data preprocessing, clustering, and visualization were conducted using the Seurat package in R. UMAP was used for dimensionality reduction and cell cluster visualization. Expression levels of SHROOM1, SHROOM2, SHROOM3, and SHROOM4 were analyzed across cell types using violin and UMAP feature plots. Pathway activity for hallmark angiogenesis and Wnt/β-catenin signaling was evaluated using the AUCell package, and enrichment scores were visualized on UMAP plots.

### 2.2 ROC analysis of SHROOM4 protein in the diagnosis of lung cancer

Receiver operating characteristic (ROC) curve analysis and testing were performed using the pROC [1.18.0] package in R to explore the sensitivity and specificity of using SHROOM4 to distinguish lung cancer patients from healthy individuals, with results visualized using ggplot2.

### 2.3 Correlation and functional enrichment analysis of *SHROOM4* in LUSC

The data was divided into high and low expression groups of the SHROOM4 gene. Correlation genes between the mRNA expression of *SHROOM4* was evaluated by Spearman’s correlation coefficient and Corrplot package in R software. The original Counts matrix of the selected public data was analyzed for differences using the DESeq2/edgeR package in R, and enrichment analysis (Gene ontology & Gene Set Enrichment Analysis, GO & GSEA) was conducted using the clusterProfiler package, with the reference gene set database from MSigDB Collection (17). The significant enrichment criterion was *P.*adjust <0.05 & FDR<0.25, and the results were visualized using the ggplot2 package.

### 2.4 Immune infiltration analysis

The GEPIA database evaluated the expression of specific markers linked to immune cell infiltration as well as the relationship between *SHROOM4* expression and patient prognosis in a variety of tumor types. The correlation between *SHROOM4* expression and the abundance of specific immune cell types, including Tcm_CD8, Tem_CD8, Treg, and others, was assessed by Spearman correlation analysis.

### 2.5 Genetic alteration analysis by cBioPortal for cancer genomics

We searched the cBioPortal for Cancer Genomics (http://cbioportal.org) database to explore the *SHROOM4* alteration frequency, CNA (copy number alteration) and mutation type in NSCLC (TCGA pancancer atlas) (18). And OS differences for NSCLC with or without *SHROOM4* genetic alteration were also presented by K-M plots with log-rank p-values via NSCLC. Moreover, the correlated-genes analysis was similarly exhibited by Spearman.

### 2.6 RNA isolation and quantitative real-time PCR

Total RNA was extracted from human lung cancer/adjacent tissue cDNA chip (Shanghai Outdo Biotech Company) using Trizol (Invitrogen, Carlsbad, CA) following the manufacturer’s instructions. Reverse transcription was performed by using the Takara Reverse Transcription Kit (Thermo Fisher Scientific). Quantitative real-time PCR was conducted by using the Takara SYBR Premix ExTaq kit and calculated by means of 2^−ΔΔCt^ methods. Related primers were designed as following: *β-actin*, F: 5’- GAAGAGCTACGAGCTGCCTGA-3’, R: 5’- CAGACAGCACTGTGTTGGCG-3’; *SHROOM4*, F: 5’-CAGCCACAAAGGGAAGAAAA-3’, R: 5’- TGTCCATCAGCCTCTCCAT-3’.

### 2.7 Assay of LUSC tissue microarray

Anti-SHROOM4 (ab121316) was purchased from Abcam (Boston, MA, USA). Human LUSC tissue microarray which contained 80 cases and 160 points was obtained from Shanghai Outdo Biotech Company and the relevant immunohistochemical (IHC) experiments and statistical analysis were completed by their technicians.

### 2.8 Statistical analysis

All statistical analysis was performed using SPSS 21.0 software (SPSS Company, Chicago, Illinois, USA) and R software. And all methods were performed in accordance with the relevant guidelines and regulations. The real-time qRT-PCR and tissue microarray results were expressed as the mean ± S.D. Student’s test and Wilcoxon signed-rank test was used to compare the expression means between different groups. *P* < 0.05 indicated a statistically significant difference.

### 2.9 Ethics statement

All samples involved in this study were obtained from Shanghai Outdo Biotech Company and passed its ethical review (ID YB M-05-01), and prior informed consent obtained from all the patients. And we confirm that all methods had been carried out in accordance with the relevant guidelines and regulations of the Declaration Helsinki.

## 3 Results

### 3.1 Expression of *SHROOM4* in lung cancer tissues

**Figure 1:**
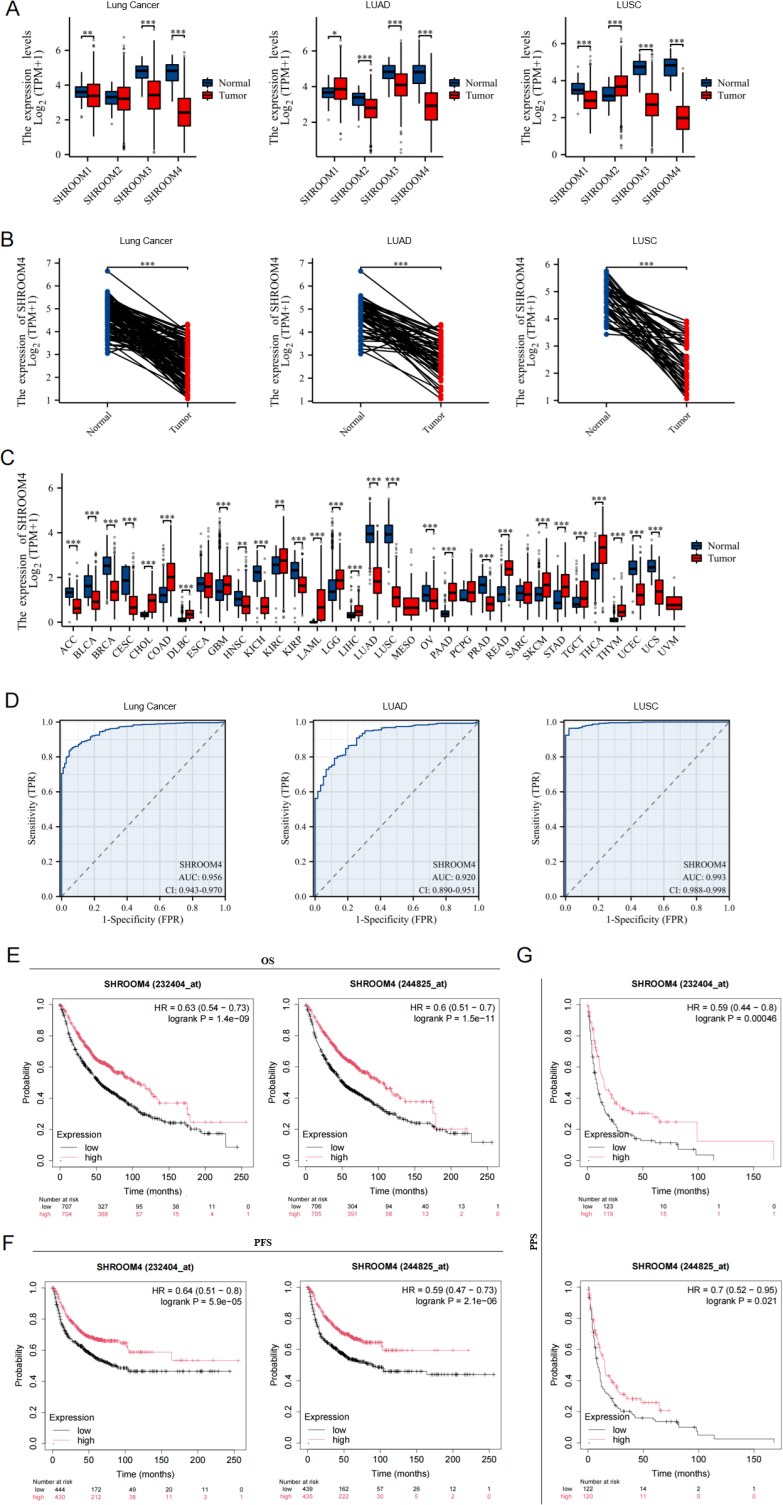
Differential expression and diagnostic value of SHROOM family genes in normal and tumor tissues. **(A)** The expression levels of SHROOM4 in lung cancer and its subtypes (LUAD: lung adenocarcinoma, LUSC: lung squamous cell carcinoma) compared with normal tissues. Boxplots show log2-transformed TPM values from TCGA datasets for SHROOM4, SHROOM2, SHROOM3, and SHROOM4. Statistical significance is denoted by *P < 0.05, **P < 0.01, ***P < 0.001. **(B)** Paired expression analysis of SHROOM4 in normal and tumor tissues in lung cancer, LUAD, and LUSC. Each line represents paired samples from the same patient, demonstrating a significant downregulation of SHROOM4 in tumor tissues (***P < 0.001). **(C)** SHROOM4 expression across various cancer types based on TCGA datasets. Boxplots compare SHROOM4 expression levels between normal and tumor tissues across 33 cancer types. Statistical significance is indicated by *P < 0.05, **P < 0.01, ***P < 0.001. **(D)** Receiver operating characteristic (ROC) curves for SHROOM4 as a diagnostic biomarker in lung cancer, LUAD, and LUSC. The area under the curve (AUC) values are shown for each cancer type, along with 95% confidence intervals (CIs). **(E)** Kaplan-Meier survival curves for overall survival (OS) based on SHROOM4 expression levels in lung cancer patients. High SHROOM4 expression is associated with improved survival outcomes. Hazard ratios (HRs) and log-rank P-values are shown. **(F)** Kaplan-Meier survival curves for progression-free survival (PFS) based on SHROOM4 expression levels. High SHROOM4 expression correlates with favorable PFS outcomes in lung cancer patients. **(G)** Kaplan-Meier survival curves for post-progression survival (PPS) based on SHROOM4 expression levels. High SHROOM4 expression is associated with better post-recurrence survival. HRs and log-rank P-values are shown.

Based on the TCGA and GTEx databases, comparison of the transcriptional expression of shroom4 family members in lung cancer tissues and normal samples indicated that mRNA expression of *SHROOM1*, *SHROOM3* and *SHROOM4* was significantly downregulated in cancer tissues. And mRNA expression of *SHROOM4* exhibited the most greatest difference in mRNA expression in lung cancer (95% CI: -2.4887–2.3058, difference -2.2626) (**Figure 1A**), especially notable in patients with LUAD (95% CI: -2.0555–1.5456, difference -1.7991) and LUSC (95% CI: -2.9622–2.4744, difference -2.7234). Compared with adjacent non-tumor tissues, paired analysis confirmed the significant downregulation of *SHROOM4* in lung cancer, specifically in LUAD and LUSC (**Figure 1B**). Furthermore, we analyzed the expression changes of *SHROOM4* across various tumors, showing that *SHROOM4* is abnormally expressed in multiple types of tumors, and it exhibited the greatest differential expression in LUSC (**Figure 1C**).

### 3.2 Diagnostic and prognostic value of *SHROOM4* in lung cancer

The diagnostic performance of *SHROOM4*, evaluated using ROC curves, indicated high sensitivity and specificity in distinguishing tumor from normal tissues, with the area under the curve (AUC) values, along with 95% confidence intervals (CI), are shown in **Figures 1D** (LC: AUC = 0.956, CI = 0.943-0.970. LUAD: AUC = 0.920, CI = 0.890-0.951. LUSC: AUC = 0.993, CI = 0.988-0.998). And we got that at a cutoff of 3.673, *SHROOM4* had a sensitivity, specificity, and accuracy of 96.4, 98 and 96.6% in LUSC, respectively. The positive value was 99.8% and the negative predictive value was 72.8%. While *SHROOM4* had a sensitivity, specificity, and accuracy of 79.2, 88.1 and 80.1% in LUAD at a cutoff of 3.787, respectively. The positive value was 98.4% and the negative predictive value was 31.7%. These findings suggested that *SHROOM4* could be a promising biomarker to differentiate lung cancer from normal tissues.

To explore the relationship between *SHROOM4* mRNA expression and survival probability in lung cancer, Kaplan-Meier curves were performed. As shown in **Figures 1E-G**, Overall survival (OS) was significantly better in patients with high *SHROOM4* expression than those with low *SHROOM4* expression, similar results were observed in Progression-free survival (PFS) and post-progression survival (PPS). In addition, patients with high *SHROOM4* expression owned higher median survival time (244825_at: OS: 103 months, PFS: 37 months, PPS: 15 months; 232404_at: OS: 103 months, PFS: 33 months, PPS: 15 months) compared with low expression group (244825_at: OS: 51 months, PFS: 13.33 months, PPS: 8.71 months; 232404_at: OS: 54.17 months, PFS: 15 months, PPS: 8 months). Collectively, the survival probability of lung cancer patients with high-level of *SHROOM4* was significantly longer than those with low-level *SHROOM4*, indicating high mRNA expression of *SHROOM4* is a biomarker of positive prognosis in lung cancer.

### 3.3 Correlation between *SHROOM4* expression and clinical features in patients with NSCLC

**Figure 2:**
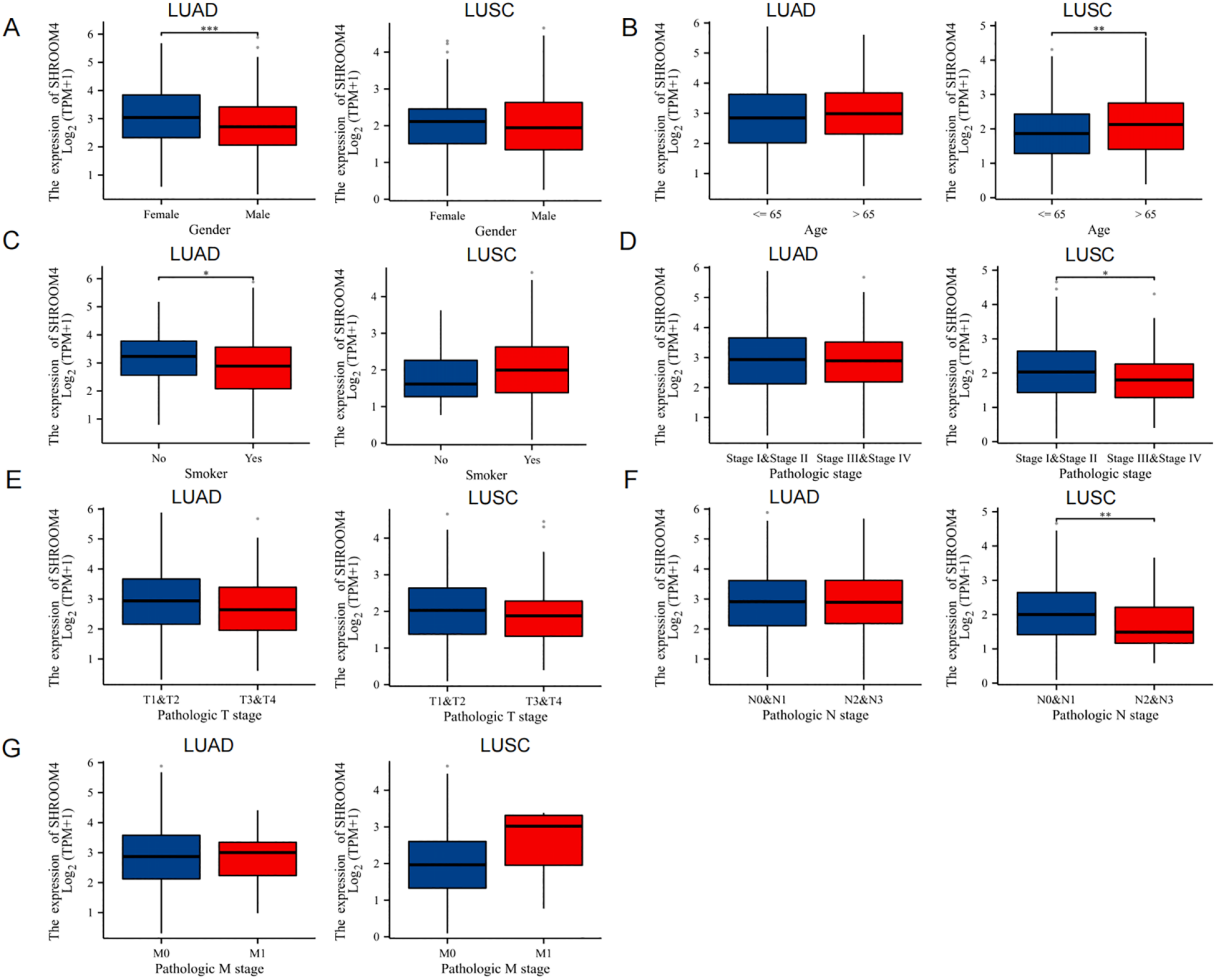
Association of SHROOM4 expression with clinical characteristics in tumor tissues. **(A)** Comparison of SHROOM4 expression levels between male and female patients in LUAD (lung adenocarcinoma) and LUSC (lung squamous cell carcinoma). SHROOM4 expression was significantly lower in male LUAD patients compared to females (***P < 0.001). **(B)** SHROOM4 expression levels in LUAD and LUSC patients stratified by age (≤65 vs. >65 years). In LUSC, SHROOM4 expression was significantly lower in patients aged >65 years (**P < 0.01). **(C)** SHROOM4 expression levels in LUAD and LUSC patients based on smoking status (Smoker vs. Non-Smoker). In LUAD, SHROOM4 expression was significantly higher in non-smokers (*P < 0.05). **(D)** SHROOM4 expression levels across different pathological stages (Stage I&II vs. Stage III&IV) in LUAD and LUSC. In LUSC, SHROOM4 expression was significantly higher in Stage III&IV patients compared to Stage I&II patients (*P < 0.05). **(E)** SHROOM4 expression levels across pathological T stages (T1&T2 vs. T3&T4) in LUAD and LUSC. No significant differences were observed. **(F)** SHROOM4 expression levels across pathological N stages (N0&N1 vs. N2&N3) in LUAD and LUSC. In LUSC, SHROOM4 expression was significantly higher in N2&N3 patients compared to N0&N1 patients (**P < 0.01). **(G)** SHROOM4 expression stratified by pathologic M stage (M0 vs. M1) in tumor tissues from two datasets (left panel: Dataset 1; right panel: Dataset 2). No significant differences are observed.

TCGA analyzed 502 LUAD samples and 539 LUSC samples with *SHROOM4* expression data. We divided patients into two groups based on the median *SHROOM4* expression level and analyzed the correlation between *SHROOM4* mRNA expression and clinical parameters by chi-square test. Our results showed that *SHROOM4* expression was correlated with gender and smoker status in LUAD, while it was correlated with age, pathologic stage and N stage in LUSC (**Figures 2A–G**). **Table 1** further presents a detailed comparison of clinical characteristics between LUAD and LUSC patients, including gender, age, smoking status, disease stage (I&II vs. III&IV), T stage, N stage, and M stage. For each clinical parameter, the number of patients, mean values, 95% confidence intervals (CI), and p-values are shown. In LUAD, significant differences were observed in gender and smoking status, while in LUSC, SHROOM4 expression showed significant correlations with age, pathologic stage, and N stage. These findings suggest that SHROOM4 expression is differentially associated with clinical features in both cancer types, highlighting its potential role in disease progression and patient stratification.

**Table 1:** Comparison of clinical characteristics between LUAD and LUSC patients.

Clinical	Subgroup	LUAD				LUSC			
		Number	Mean	95%CI	*p* value	Number	Mean	95%CI	*p* value
Gender	Female	289	3.049	-0.54164 – -0.18617	6.6e-05	131	2.0617	-0.25063 – 0.1243	0.5142
	Male	250	2.6851			371	2.0178		
Age	<= 65	257	2.8013	-0.017093 – 0.35008	0.0754	191	1.889	0.055301 – 0.39313	0.01
	> 65	263	2.9677			302	2.1141		
Smoker	Yes	453	2.8391	0.038336 – 0.55372	0.0244	472	2.0417	-0.64613 – 0.1644	0.2796
	No	77	3.1352			18	1.8109		
Stage	I & II	421	2.8875	-0.2692 – 0.17572	0.6799	407	2.0711	-0.42063 – -0.01525	0.0369
	III & IV	110	2.8407			91	1.871		
T Stage	T1 & T2	468	2.9025	-0.46239 – 0.079049	0.1649	408	2.051	-0.33651 – 0.06676	0.189
	T3 & T4	68	2.7108			94	1.9347		
N Stage	N0 & N1	447	2.8552	-0.24526 – 0.27356	0.9147	451	2.061	-0.65925 – -0.13495	0.0032
	N2 & N3	76	2.8693			45	1.6733		
M Stage	M0	365	2.8175	-0.49021 – 0.36142	0.7664	412	2.0234	-0.21519 – 1.3582	0.1386
	M1	25	2.7532			7	2.5298		

This table compares the clinical characteristics and prognostic factors between LUAD and LUSC patients. For each clinical variable, the number of patients, mean values, 95% confidence intervals (CI), and p-values are provided. The table includes data on gender, age, smoking status, disease stage (I&II vs. III&IV), T stage, N stage, and M stage. The p-values indicate statistical significance, with values less than 0.05 suggesting notable differences between the two cancer types in specific variables, such as age and smoking status, while other variables show no significant differences.

### 3.4 Expression of SHROOM4 protein and phosphorylation levels in lung cancer

**Figure 3:**
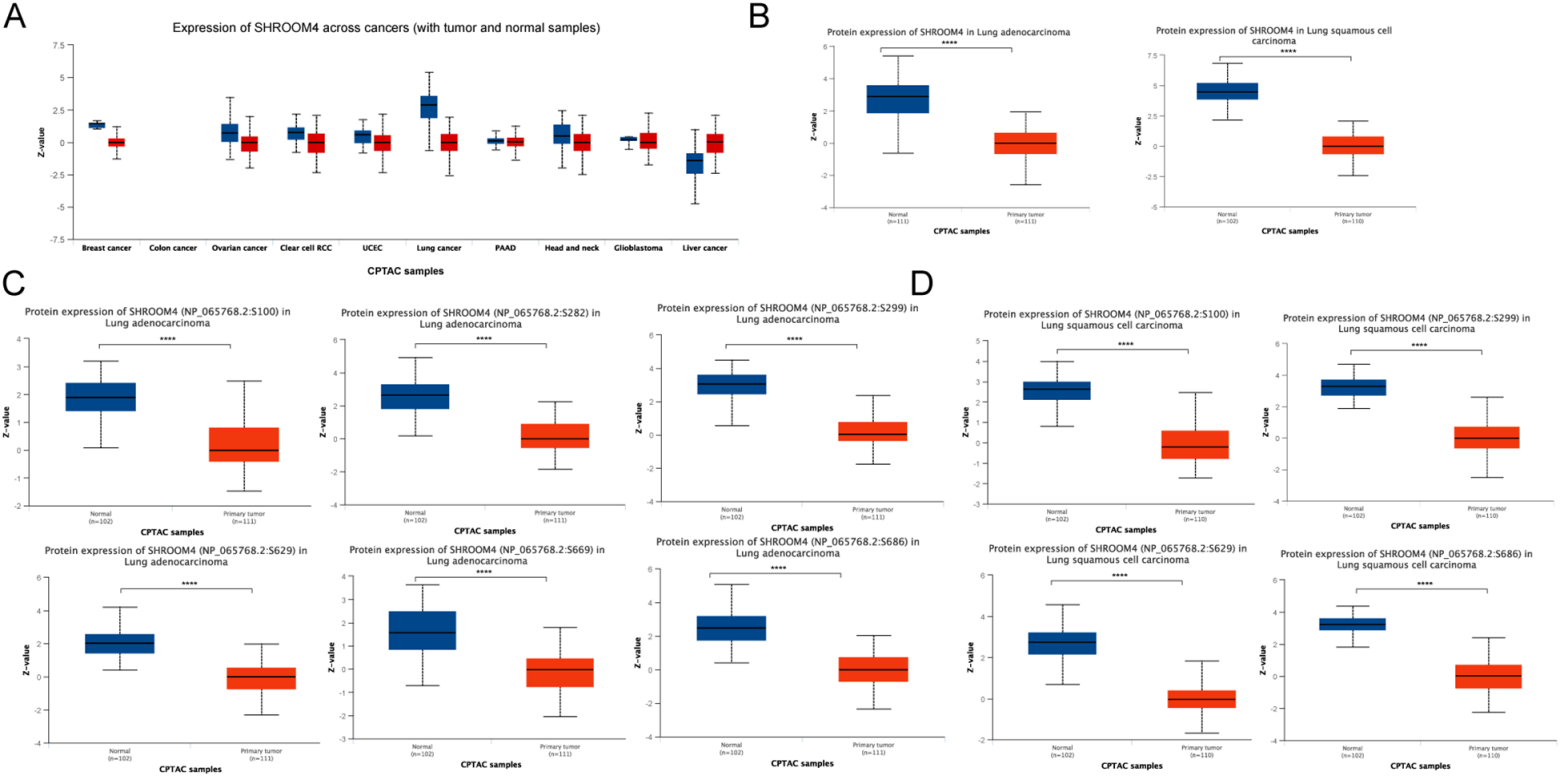
SHROOM4 protein expression and phosphorylation levels. **(A)** Total SHROOM4 protein expression levels across various cancer types (e.g., breast cancer, colon cancer, ovarian cancer, clear cell RCC, UCEC, lung cancer, glioblastoma, and liver cancer) compared with normal tissues. Boxplots show Z-scores of SHROOM4 protein expression, with blue representing normal tissues and red representing tumor tissues. Significant differences in expression levels are observed across multiple cancer types. **(B)** Total SHROOM4 protein expression in lung adenocarcinoma (LUAD) and lung squamous cell carcinoma (LUSC). Boxplots compare SHROOM4 protein levels between normal (blue) and primary tumor (red) tissues. SHROOM4 expression is significantly downregulated in tumor tissues in both LUAD and LUSC (****P < 0.0001). **(C)** Phosphorylation levels of SHROOM4 at specific sites (S100, S282, S299, S629, S669, and S686) in LUAD. Boxplots display Z-scores for each phosphorylation site, comparing normal (blue) and primary tumor (red) tissues. Significant reductions in phosphorylation levels at these sites are observed in LUAD tumor tissues (****P < 0.0001). **(D)** Phosphorylation levels of SHROOM4 at specific sites (S100, S299, S629, and S686) in LUSC. Boxplots display Z-scores for each phosphorylation site, comparing normal (blue) and primary tumor (red) tissues. Phosphorylation levels are significantly reduced at these sites in LUSC tumor tissues (****P < 0.0001).

In our study using proteomics data from the CPTAC, we found that SHROOM4 expression is generally downregulated across various cancers when compared to normal tissues (**Figure 3A**). This reduction was notably pronounced in lung cancer, prompting a further investigation into its expression across different lung cancer subtypes. Specifically, LUAD and LUSC patient samples revealed a significant downregulation of SHROOM4 compared to adjacent normal tissues (**Figure 3B**). Additionally, the protein expression of SHROOM4 with phosphorylation sites at the S100, S299, S629 and S686 were viewed the lower level in LUAD and LUSC patients, while its sites at the S282 and S669 were only captured in LUAD patients (**Figures 3C, D**). The *p* value all less than 0.05. These results suggest a link between the reduced expression of SHROOM4 in lung cancer and alterations in protein modification.

### 3.5 Single-cell RNA-seq analysis of shroom family gene expression in NSCLC

**Figure 4:**
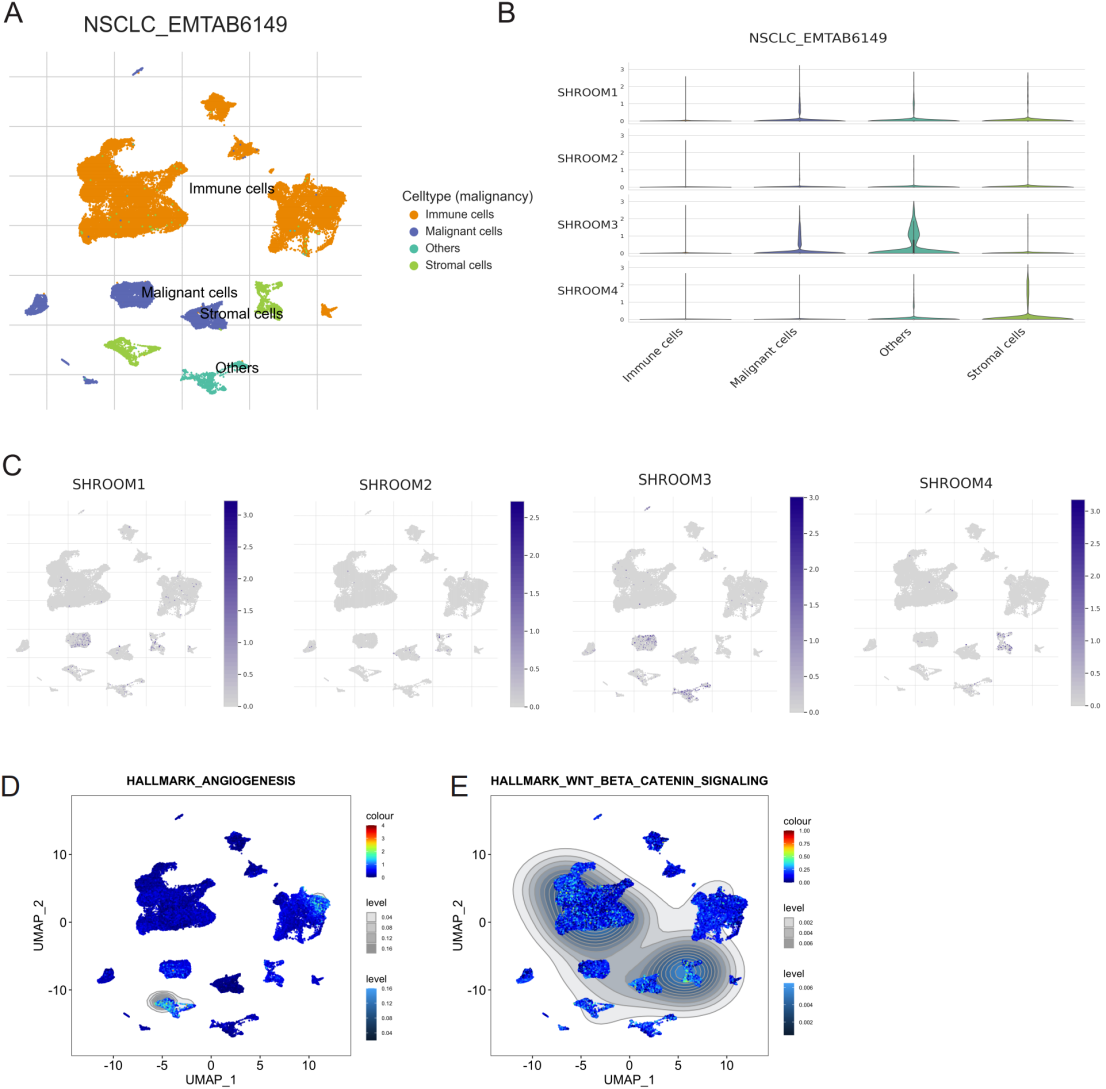
Single-cell RNA-seq analysis of SHROOM family gene expression in non-small cell lung cancer (NSCLC). **(A)** UMAP plot showing the clustering of different cell types in the NSCLC_EMTAB6149 dataset. Cells are color-coded based on their type: immune cells (orange), malignant cells (blue), stromal cells (green), and others (gray). **(B)** Violin plots depicting the expression levels of SHROOM1, SHROOM2, SHROOM3, and SHROOM4 across different cell types (immune cells, malignant cells, others, stromal cells) in the NSCLC_EMTAB6149 dataset. **(C)** UMAP plots showing the expression of SHROOM1, SHROOM2, SHROOM3, and SHROOM4 at single-cell resolution in the NSCLC_EMTAB6149 dataset. Color intensity represents the expression level, with darker blue indicating higher expression. **(D)** UMAP plot visualizing the enrichment of the Hallmark Angiogenesis gene set across the cell clusters in the NSCLC_EMTAB6149 dataset. **(E)** UMAP plot visualizing the enrichment of the Hallmark Wnt/Beta-Catenin Signaling gene set across the cell clusters in the NSCLC_EMTAB6149 dataset.

Single-cell RNA-seq analysis in the NSCLC_EMTAB6149 dataset revealed distinct clustering of cell types, including immune cells, malignant cells, and stromal cells (**Figure 4A**). Among these, *SHROOM4* is predominantly expressed in stromal cells, as highlighted by the violin plots (**Figure 4B**), where its expression surpasses other Shroom family members. UMAP feature plots (**Figure 4C**) further confirm the spatial distribution of *SHROOM4*, with its expression concentrated in stromal cells, reinforcing its potential role in the tumor microenvironment. Pathway enrichment analysis (**Figures 4D, E**) links *SHROOM4* expression to hallmark angiogenesis and Wnt/β-catenin signaling pathways, both of which are key drivers of tumor progression and microenvironment remodeling.

### 3.6 Functional analysis of *SHROOM4* gene in NSCLC

**Figure 5:**
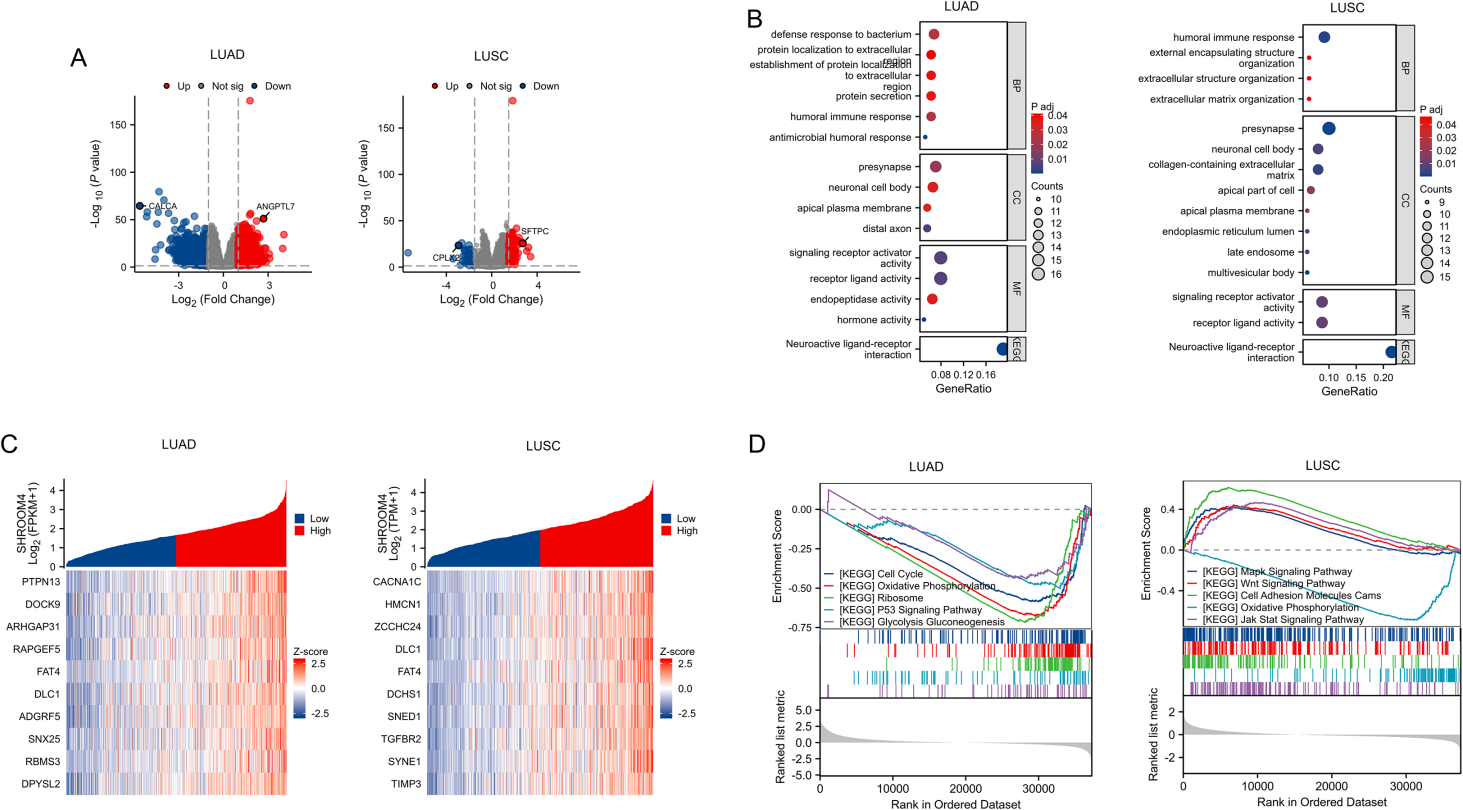
Differential expression analysis, functional enrichment, and pathway activity of SHROOM4 in LUAD and LUSC. **(A)** Volcano plots showing differentially expressed genes (DEGs) between high and low SHROOM4 expression groups in LUAD (left) and LUSC (right). Each dot represents a gene, with red dots indicating significantly upregulated genes (Log2 Fold Change > 1, adjusted P < 0.05) and blue dots indicating significantly downregulated genes (Log2 Fold Change < -1, adjusted P < 0.05). Notable genes such as ANGPTL7 (LUAD) and SFTPC (LUSC) are highlighted. **(B)** Functional enrichment analysis of DEGs in LUAD (left) and LUSC (right) categorized by GO terms for biological processes (BP), cellular components (CC), molecular functions (MF), and KEGG pathways (KEGG). Dot size indicates the number of enriched genes, and color represents the adjusted P-value. Top enriched terms include immune response and extracellular matrix organization in both LUAD and LUSC. **(C)** Heatmap of top 15 differentially expressed genes associated with SHROOM4 expression in LUAD (left) and LUSC (right). Genes are ranked by their Z-scores. Patients are divided into high (red) and low (blue) SHROOM4 expression groups, showing distinct gene expression profiles. **(D)** Gene set enrichment analysis (GSEA) of KEGG pathways in LUAD (left) and LUSC (right) comparing high and low SHROOM4 expression groups. Pathways such as "Cell Cycle," "Oxidative Phosphorylation," and "Ribosome" are significantly enriched in LUAD, while "Wnt Signaling Pathway," "Jak-STAT Signaling Pathway," and "MAPK Signaling Pathway" are enriched in LUSC.

We conducted differential expression analysis between the low and high *SHROOM4* in LUAD & LUSC and only protein-coding genes were retained. As shown in **Figure 5A**, a total of 530 differential genes in LUAD, including 168 up-regulated genes and 362 down-regulated genes, were selected according to the |log2FC| >1.5 & *p* < 0.05 screening conditions (**Figure 5A**, **Supplementary Data 1**). And a total of 153 differential genes in LUSC, including 114 up-regulated genes and 39 down-regulated genes. *CALCA* and *ANGPTL7* were specifically labeled in LUAD, while *SFTPC* and *CPLX2* were labeled in LUSC. Next, above differential genes were studied for GO, KEGG pathway and GSEA analysis to get the function of *SHROOM4*. As displayed in **Figure 5B** and **Supplementary Tables S1, S2**, these genes were involved in humoral immune response, protein localization to extracellular, endopeptidase activity and other processes in LUAD. In contrast, differential expression genes were participated in humoral immune response, extracellular matrix organization, collagen-containing extracellular matrix, presynapse and others in LUSC. Meanwhile, cell cycle, oxidative phosphorylation, ribosome, P53 and glycolysis gluconeogenesis pathways were significantly enriched in LUAD, while MAPK, WNT, JAT-STAT, cell adhesion molecules cams and oxidative phosphorylation signaling pathways were significantly enriched in LUSC (**Figure 5D**, **Supplementary Tables S5, S6**). Lastly, SHROOM4-related genes were picked according to the |Cor| >0.5 & *p* < 0.05 and top 10 genes were displayed in heat-map and **Supplementary Data 2** (**Figure 5C**, **Supplementary Tables S3, S4**). Interestingly, *PTPN13* was positively associated with *SHROOM4* in LUAD and CACNA1C was positively correlated with *SHROOM4* in LUSC. These results might assistant to further understand NSCLC patho-physiological mechanisms.

### 3.7 *SHROOM4* expression correlated with immune infiltration levels in NSCLC

**Figure 6:**
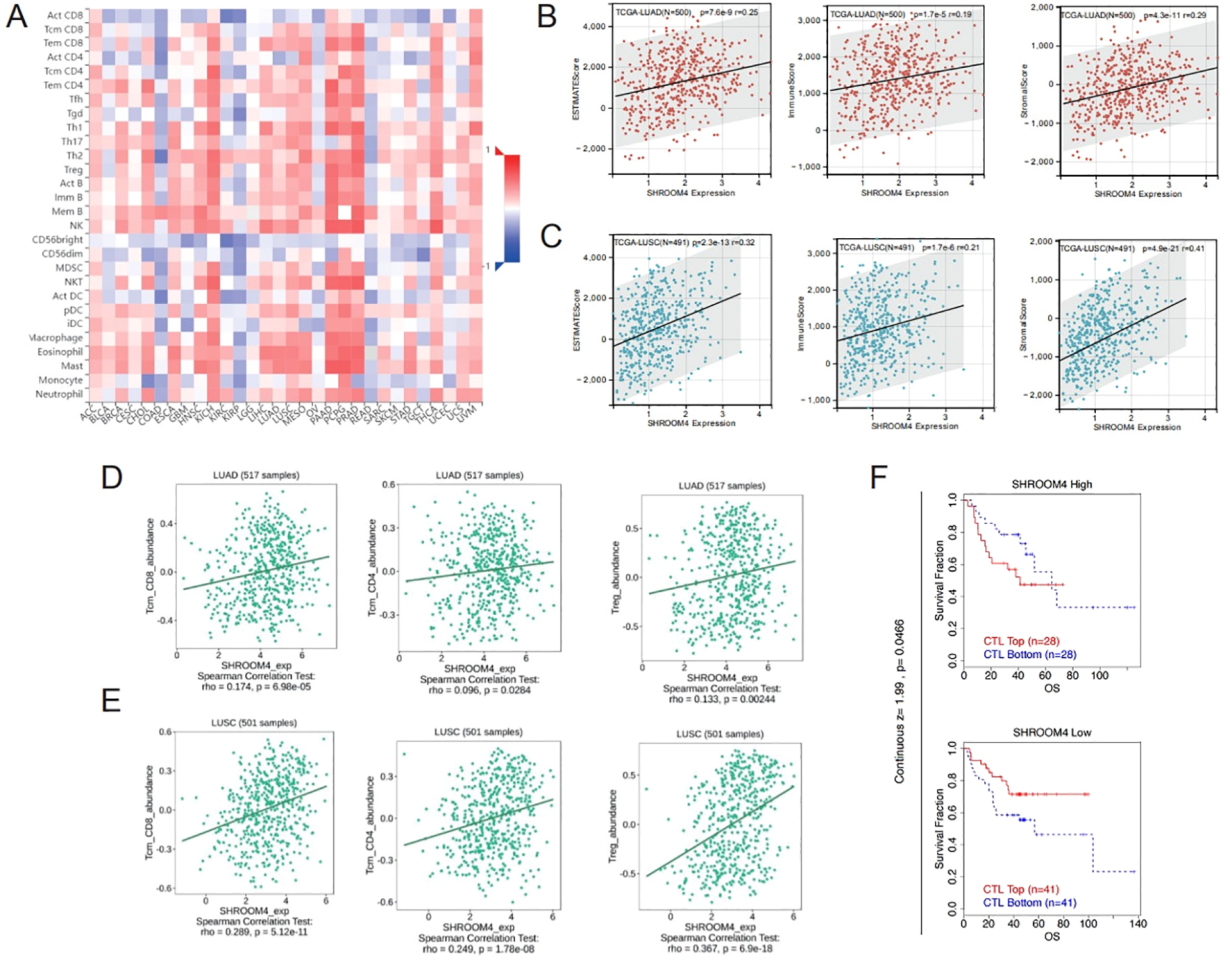
Correlation between SHROOM4 expression and immune infiltration in lung cancer. **(A)** Heatmap showing the correlation between SHROOM4 expression and various immune cell types in lung cancer. The color scale represents the Z-score, with red indicating higher correlation and blue indicating lower correlation. **(B)** Scatter plots showing the correlation between SHROOM4 expression and ESTIMATE, StromalScore and ImmuneScore scores in LUAD from the TCGA dataset. Spearman correlation coefficients (r) and p-values are indicated. **(C)** Scatter plots showing the correlation between SHROOM4 expression and ESTIMATE, StromalScore and ImmuneScore scores in LUSC from the TCGA dataset. Spearman correlation coefficients (r) and p-values are indicated. **(D)** Scatter plots showing the correlation between SHROOM4 expression and Tcm_CD8, Tem_CD4 and Treg abundance in LUAD. Spearman correlation coefficients (r) and p-values are indicated. **(E)** Scatter plots showing the correlation between SHROOM4 expression and Tcm_CD8, Tem_CD4 and Treg abundance in LUSC. Spearman correlation coefficients (r) and p-values are indicated. **(F)** Kaplan-Meier survival curves comparing overall survival (OS) between high and low SHROOM4 expression groups in lung cancer, stratified by CTL (Cytotoxic T Lymphocyte) abundance. Patients are divided into top and bottom CTL groups based on SHROOM4 expression. Log-rank test p-value is shown.

The presence of lymphocytes within a tumor is a significant factor that can independently forecast the duration of survival and the likelihood of lymph node involvement in individuals with cancer. A comprehensive heatmap displayed correlations between *SHROOM4* expression and various immune cell types (**Figure 6A**). *SHROOM4* expression showed significant correlations with immune infiltration scores in lung cancer. Positive correlations were observed between *SHROOM4* expression and ESTIMATE, Immune, and Stromal scores in LUAD and LUSC (**Figures 6B, C**). Further analysis indicated positive associations with specific immune cell types, including Tcm_CD8, Tcm_CD4, and Treg abundances (**Figures 6D, E**). These results clearly show that *SHROOM4* might attract immune cells to the tumor microenvironment (TME) in LUAD, and LUSC, particularly on, CD8 + T cells, CD4 + T cells, and Treg T cells. Survival analysis based on *SHROOM4* expression and CTL abundance suggested a potential impact on overall survival (**Figure 6F**). These data indicated that *SHROOM4* was closely associated with the infiltration of immune cells, indicating that *SHROOM4* plays an important role in NSCLC partly because of immune infiltration.

*SHROOM4* may play a specific role in immune infiltration in NSCLC.

### 3.8 Genetic alterations of *SHROOM4* in NSCLC

**Figure 7:**
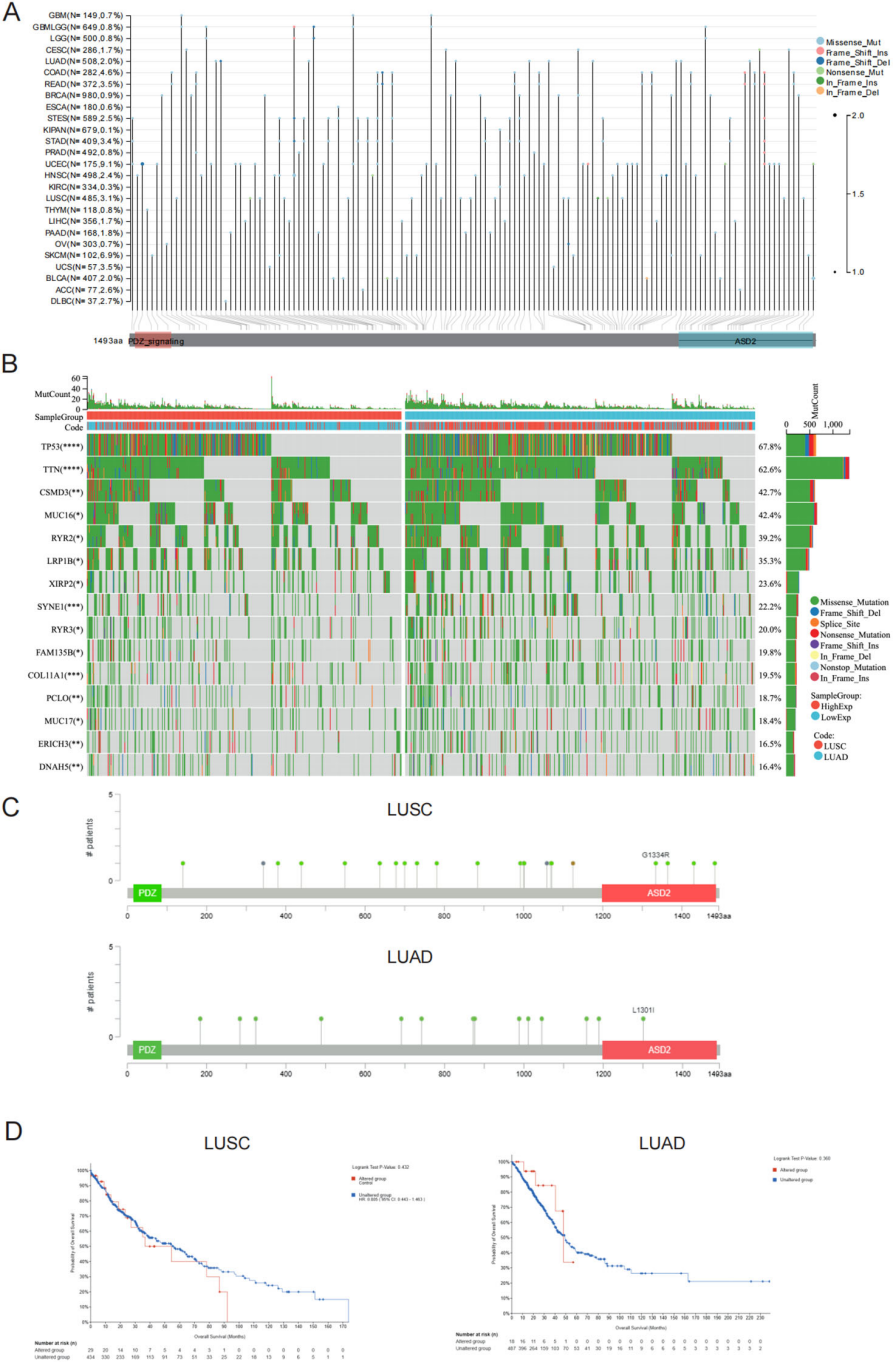
Genetic alterations and clinical relevance of SHROOM4 in lung cancer. **(A)** Lollipop plot displaying the mutation distribution of SHROOM4 across multiple cancer types from TCGA datasets. Each dot represents a mutation, and its position indicates the location of the mutation along the SHROOM4 protein sequence (1–1493 amino acids). The type of mutation is indicated by color: missense mutations (blue), nonsense mutations (red), frameshift insertions/deletions (green), and in-frame insertions/deletions (purple). Functional domains PDZ and ASD2 are highlighted, with notable mutations such as G1334R and L1301I marked. **(B)** Oncoplot of SHROOM4 mutations in LUAD (right) and LUSC (left) showing the top 20 co-mutated genes. Each column represents a patient, and each row represents a gene. Mutation types are color-coded, with missense mutations being the most frequent. The top bar graph shows the number of mutations per patient, and the side bar graph shows the mutation frequency for each gene. Mutation frequencies for SHROOM4 are stratified by expression levels (high vs. low) and tumor subtype (LUAD vs. LUSC). **(C)** Protein mutation plots for SHROOM4 in LUAD (bottom) and LUSC (top), showing the frequency of mutations across the protein sequence. Green dots represent mutation sites, and the height of the dot indicates the number of patients with the mutation. Functional domains PDZ and ASD2 are highlighted, with notable mutations such as G1334R in LUSC and L1301I in LUAD. **(D)** Kaplan-Meier survival analysis for overall survival (OS) in LUSC (left) and LUAD (right) patients with SHROOM4 mutations. Patients are stratified into altered and unaltered groups. Survival curves show that SHROOM4 mutations are associated with poorer prognosis in LUSC but not in LUAD. Log-rank P-values are provided.

Our findings underscore a significant link between *SHROOM4* and lung cancer, prompting us to delve deeper into its genetic underpinnings. Using the cBioPortal for Cancer Genomics, *SHROOM4* mutations were observed across various cancer types, with different mutation types including missense, frame shift, nonsense, and in-frame mutations (**Figure 7A**). And the genetic alteration frequency of *SHROOM4* was 3.1% in LUSC but 2% in LUAD. The oncoplot displayed the mutation profiles of *SHROOM4* and other frequently mutated genes in LUAD & LUSC, highlighting the mutation burden in these cancers (**Figure 7B**). And we got that the higher expression may associate with the mutation possibility of *TP53* in LUAD and LUSC (*p* < 0.0001), which the major mutation types are missense, frame-shift-deletion, nonsense and splice site mutation. A detailed mutation analysis of *SHROOM4* revealed specific mutation sites along the protein sequence in **Figure 7C**. As shown as in **Figure 7D**, LUAD & LUSC cases with altered *SHROOM4* did not observe better prognosis in OS (*p* = 0.432 and 0.360).

### 3.9 qRT-PCR and tissue microarray to further validate the mRNA and protein expression of *SHROOM4* in LUSC

**Figure 8:**
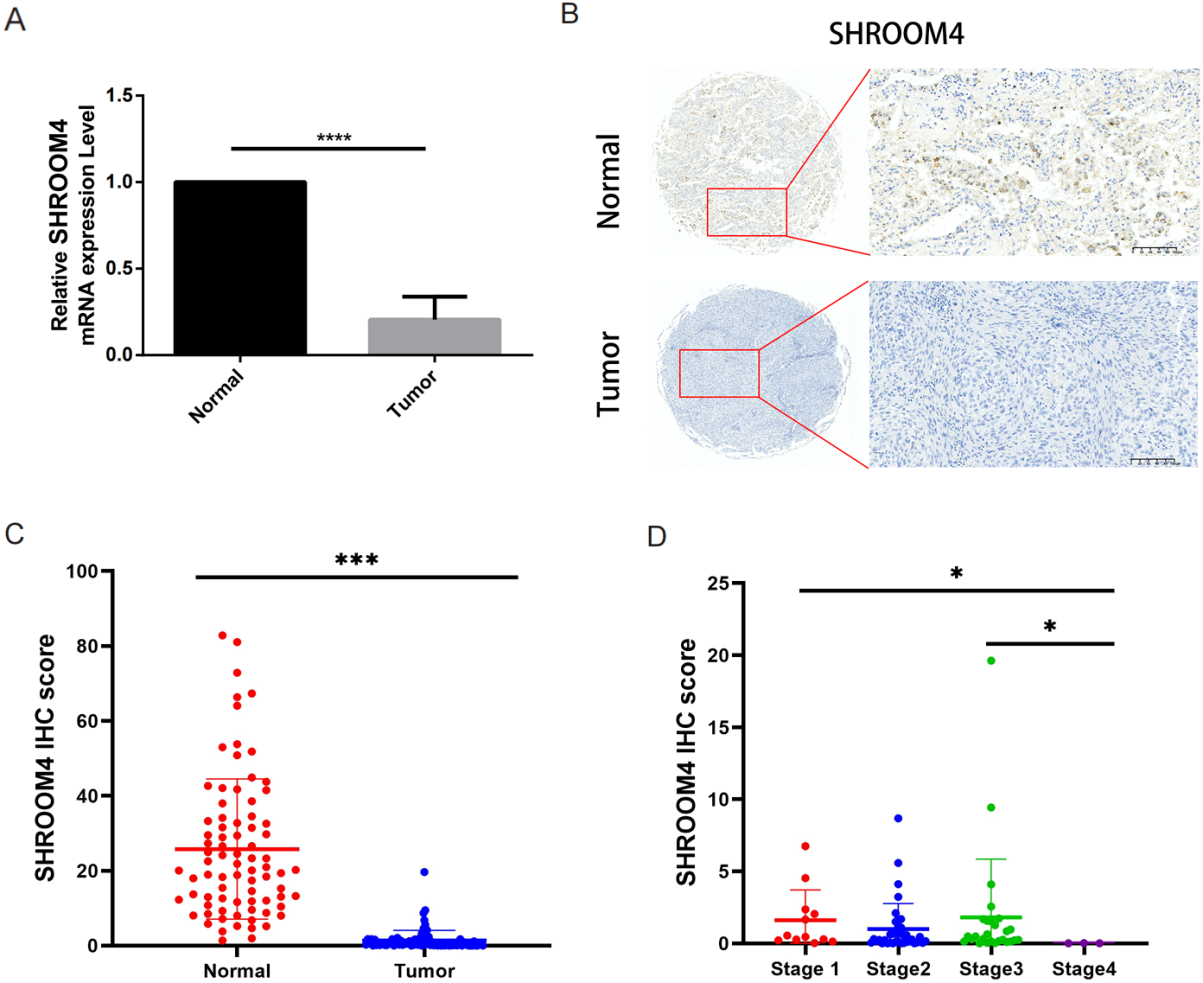
SHROOM4 expression analysis in tumor and normal tissues. **(A)** Relative mRNA expression levels of SHROOM4 in paired normal and tumor tissues from patients, measured by qRT-PCR. SHROOM4 expression is normalized using the 2^-ΔΔCT method. Each bar represents an individual sample, showing significantly lower SHROOM4 mRNA levels in tumor tissues compared to normal tissues (****p < 0.0001). **(B)** Representative immunohistochemistry (IHC) staining images of SHROOM4 in normal (top) and tumor (bottom) tissues. The normal tissue shows strong SHROOM4 protein expression, while the tumor tissue exhibits a significant loss of SHROOM4 expression. Scale bar: 100 μm. **(C)** Violin plot comparing IHC H-scores for SHROOM4 expression between normal and tumor tissues. SHROOM4 expression is significantly reduced in tumor tissues compared to normal tissues (****p < 0.0001). **(D)** Comparison of SHROOM4 IHC H-scores across different tumor pathological stages (Stages I–IV) (*p < 0.05).

For verified the expression level of *SHROOM4* in LUSC, we utilized qRT-PCR and tissue microarray assay based on IHC to conduct. As displayed in **Figure 8A**, *SHROOM4* mRNA expression was down-regulated in the LUSC tumors (N = 15) compared with normal adjacent tissues (N = 15, p < 0.0001). On the other hand, the protein levels of SHROOM4 in LUSC tumor tissues were significantly lower than those in adjacent normal tissues (**Figures 8B, C**, difference -24.45 ± 2.117, *p* < 0.0001). Subsequently, we divided the tumor tissues into a high IHC score group and a low IHC score group, then analyzed the IHC scores of SHROOM4 in these two groups. As shown in **Figure 8D**, the IHC scores of SHROOM4 in stage 4 were significantly lower than those in clinical stage 1 & 3 (*p =* 0.031), indicating that SHROOM4 protein levels negatively correlate with clinical stage. Combing with the aforementioned survival analysis, SHROOM4 is down-expressed and may play a protective role in LUSC.

## 4 Discussion

The role of *SHROOM4* played in NSCLC remains poorly understood. Consequently, this study aims to provide a comprehensive investigation into the expression of *SHROOM4* in NSCLC and its correlation with clinical outcomes and molecular features, with particular emphasis on lung adenocarcinoma (LUAD) and lung squamous cell carcinoma (LUSC).

Our findings indicate a significant downregulation of *SHROOM4* at both the mRNA and protein levels in LUAD and LUSC tissues, relative to adjacent non-tumor tissues. Furthermore, elevated *SHROOM4* expression is associated with favorable overall survival (OS), disease-free survival (FPS), and progression-free survival (PPS) in lung cancer, exhibiting high diagnostic accuracy, especially in LUSC. Phosphorylation analyses revealed decreased phosphorylation at multiple sites (S100, S299, S629, S686, S282, and S669) in LUAD, while similar alterations were observed in LUSC, with the exception of S282 and S699. Notably, *SHROOM4* mRNA expression was found to correlate with gender and smoking status in LUAD patients, whereas in LUSC, it was associated with age, pathological stage, and N stage. Given that the most pronounced differential expression of *SHROOM4* was observed in LUSC among various cancer types, we further validated the reduced mRNA and protein expression of *SHROOM4* in LUSC through quantitative reverse transcription polymerase chain reaction (qRT-PCR) and immunohistochemistry (IHC). Notably, IHC scores in advanced clinical stages of LUSC were significantly lower compared to early stages, suggesting a relationship with cancer progression. In summary, *SHROOM4* may serve as a protective molecular marker and prognostic predictor in NSCLC, and it holds potential as a promising diagnostic biomarker for NSCLC patients.

The interactions of cells in tumor microenvironment (TME, composed of immune cells, stromal cells, endothelial cells, secreted cytokines, extracellular matrix, vascular networks and so on), not only between tumor cells and stromal cells but also between stromal cells, results in a constantly changing TME during tumor progression, resulting in a dynamic process that has an important role in tumor development (19–21). Single-cell RNA sequencing (RNA-seq) analyses have provided valuable insights into the cell-type-specific expression of Shroom family genes. Notably, *SHROOM4* is predominantly expressed in stromal cells, while *SHROOM3* is more highly expressed in malignant tumor cells. Additionally, key pathways such as angiogenesis and the Wnt/β-catenin signaling cascade are enriched in lung cancer, further underscoring the complexity of the TME in this context.

In this study, *ANGPTL7*, a molecule involved in modulating angiogenesis and maintaining vascular integrity and permeability (22), was identified as negatively correlated with *SHROOM4* in LUAD. ANGPTL7 protein is secreted and localizes to the perinecrotic zone as a tumor-specific factor, with a partial association to the exosomal fraction. This protein exerts a pro-angiogenetic effect on human differentiated endothelial cells by stimulating their proliferation, motility, invasiveness, and capability to form capillary-like networks (23, 24). The loss of SHROOM4 relieves its inhibition on the pro-angiogenic factor ANGPTL7, thereby accelerating the progression and metastasis of LUAD. In contrast, *SFTPC*, a marker gene of alveolar type 2 (AT2) cells (25), was found and exhibited high-expressed in LUSC. Abnormal AT2 cells are known to be the origin of NSCLC, and the dysregulation of SFTPC may drive epithelial-mesenchymal transition (EMT) in these cells (26, 27). A growing body of evidence links the Wnt/β-catenin signaling pathway with EMT, influencing tumor growth and metastasis in NSCLC (28, 29). *SHROOM4* likely controls tumor progression and metastasis via the Wnt/β-catenin signaling pathway through the modulation of *SFTPC*. The loss of *SHROOM4* further promotes tumor progression and metastasis by upregulating the expression of SFTPC, thereby enhancing the Wnt/β-catenin signaling pathway. The findings suggest that different members of the *SHROOM* family may have distinct roles in various cell types and biological functions within the tumor, thereby influencing the TME and tumor progression.

Furthermore, we also disclosed that *SHROOM4* may be part of a broader regulatory network influencing TME interactions and extracellular matrix remodeling and immune response in LUAD or LUSC. Moreover, we observed *PTPN13* may be co-expressed with *SHROOM4* in LUAD, and while *CACNA1C* was found to co-expressed with *SHROOM4* in LUSC. *PTPN13*, a tumor suppressor and localizing to specific sites along the mitotic spindle, regulates cellular proliferation and invasive characteristics in multiple epithelial cells in tumors through accommodating cell circle (30, 31). Low expression of *SHROOM4* reduces the tumor-suppressive effect of PTPN13, leading to uncontrolled cell cycle progression in LUAD. On the other hand, *CACNA1C*, a subunit of voltage-gated calcium channels Cav1.2, has been implicated in regulating cell-matrix adhesion, collagen fibril organization, cell adhesion, cellular response to amino acid stimulus, and negative regulation of cell proliferation (32–34). CACNA1C has been confirmed to be associated with tumor metastasis and invasion. Our results also reveal that the low expression of *SHROOM4* promotes LUSC progression by inhibiting CACNA1C (35, 36).

In the tumor microenvironment (TME), the immune system plays a double-edged sword role. On one hand, the immune system can recognize and eliminate cancer cells through cytotoxic T cells and natural killer (NK) cells, thereby inhibiting tumor growth. On the other hand, tumors can evade immune attack through “immune escape” mechanisms and hijack immune cells (such as macrophages and myeloid-derived suppressor cells) to create a favorable environment that promotes tumor growth, angiogenesis, and metastasis (37, 38). Given the critical role of TME in mediating cancer progression and the fact that tumor-infiltrating immune cells are an integral component of TME, we sought to investigate the relationship between *SHROOM4* and immune infiltration in NSCLC. The results showed that *SHROOM4* expression was positively correlated with immune infiltration scores, especially in Tcm_CD8, Tcm_CD4, and Treg cells, indicating that *SHROOM4* might attract immune cells to the tumor microenvironment. Here, we also linked the survival possibility to the relationship between cytotoxic T lymphocytes (CTL) abundance and *SHROOM4* expression and addicted that a higher abundance of CTLs may have an anti-tumor effect, particularly in the group of patients with low *SHROOM4* expression in lung cancer. In conclusion, our results indicate that low expression of *SHROOM4* in LUAD and LUSC leads to the exhaustion of immune cells, impairing the body’s ability to effectively target and kill tumor cells. The growing field of immune-based therapies, which aim to enhance CTL-mediated tumor cell killing by targeting the TME, has shown promising results in various human malignancies (39, 40). However, advanced NSCLC patients are resistant to many targeted therapeutic drugs and have poor prognosis. Therefore, our findings may provide new insights into NSCLC immunotherapy, highlighting *SHROOM4* as a potential therapeutic target for modulating immune responses within the TME.

Genetic alterations have been implicated in the development and progression of various cancers (41–43). Here, we examined the genetic alterations of *SHROOM4* and found that the frequency of *SHROOM4* alterations is 2% in LUAD and 3.1% in LUSC. And the mutations are in key phosphorylation sites - ASD2 domain. In additionally, results of KM plotter showed no significantly statistical differences in OS of LUAD & LUSC with and without *SHROOM4* alterations.

Although our outcomes may provide new insights into the correlation between *SHROOM4* and NSCLC, certain limitations were noted in this research. Due to data downloaded directly from public databases that may cause sample bias, further experimental validation is required to elucidate the biological functions of *SHROOM4 in vitro* and *in vivo*.

## 5 Conclusions

All in all, our comprehensive analysis demonstrates that *SHROOM4* is significantly downregulated in NSCLC and is associated with clinical outcomes, genetic alterations, immune infiltration, angiogenesis, stromal cells and extracellular matrix. Meanwhile, *SHROOM4* could serve as both a diagnosis and prognostic biomarker in NSCLC, particularly in LUSC. Furthermore, *SHROOM4* appears to regulate the tumor microenvironment (TME) through mechanisms such as angiogenesis and cell cycle modulation mediated by *ANGPTL7* and *PTPN13* in LUAD, thereby influencing cancer progression. In LUSC, *SHROOM4* may modulate the TME via the Wnt/β-catenin signaling pathway, potentially involving *SFTPC* and *CACNA1C*. However, further functional studies are required to elucidate the precise mechanisms by which *SHROOM4* influences lung cancer progression and to confirm its clinical applicability in the management of NSCLC.

## Data Availability

The raw data supporting the conclusions of this article will be made available by the authors, without undue reservation.
